# Self-nanoemulsifying system improves oral absorption and enhances anti-acute myeloid leukemia activity of berberine

**DOI:** 10.1186/s12951-018-0402-x

**Published:** 2018-10-05

**Authors:** Jieping Li, Li Yang, Rui Shen, Li Gong, Zhiqiang Tian, Huarong Qiu, Zhe Shi, Lichen Gao, Hongwu Sun, Guangsen Zhang

**Affiliations:** 1 Department of Hematology, Changsha Central Hospital, Changsha, 410004 Hunan People’s Republic of China; 2 Department of Clinical Laboratory, The Third Affiliated Hospital, Chongqing Medical University, Chongqing, 401120 People’s Republic of China; 3 Army Military Medical University of Chinese PLA, Chongqing, 400038 People’s Republic of China; 4 Air Force Military Medical University of Chinese PLA, Xi’an, 710000 Shanxi People’s Republic of China; 5 Department of Pharmacy, Cancer Institute, Phase I Clinical Trial, Changsha Central Hospital, Changsha, 410004 Hunan People’s Republic of China; 6 Department of Hematology, The Second Xiangya Hospital of Central South University, Changsha, 410008 Hunan People’s Republic of China

**Keywords:** Berberine, Self-nanoemulsifying system, Acute myeloid leukemia, MV4-11, Caco-2 monolayer transport

## Abstract

**Background:**

Recently, we found that berberine (BBR) exerts anti-acute myeloid leukemia activity, particularly toward high-risk and relapsed/refractory acute myeloid leukemia MV4-11 cells in vitro. However, the poor water solubility and low bioavailability observed with oral BBR administration has limited its clinical use. Therefore, we design and develop a novel oil-in-water self-nanoemulsifying system for BBR (BBR SNE) to improve oral bioavailability and enhance BBR efficacy against acute myeloid leukemia by greatly improving its solubility.

**Results:**

This system (size 23.50 ± 1.67 nm, zeta potential − 3.35 ± 0.03 mV) was prepared with RH40 (surfactant), 1,2-propanediol (co-surfactant), squalene (oil) and BBR using low-energy emulsification methods. The system loaded BBR successfully according to thermal gravimetric, differential scanning calorimetry, and Fourier transform infrared spectroscopy analyses. The release profile results showed that BBR SNE released BBR more slowly than BBR solution. The relative oral bioavailability of this novel system in rabbits was significantly enhanced by 3.41-fold over that of BBR. Furthermore, Caco-2 cell monolayer transport studies showed that this system could help enhance permeation and prevent efflux of BBR. Importantly, mice with BBR SNE treatment had significantly longer survival time than BBR-treated mice (*P *<* 0.001*) in an MV4-11 engrafted leukemia murine model.

**Conclusions:**

These studies confirmed that BBR SNE is a promising therapy for acute myeloid leukemia.

**Electronic supplementary material:**

The online version of this article (10.1186/s12951-018-0402-x) contains supplementary material, which is available to authorized users.

## Background

Acute myeloid leukemia (AML) is the most common acute leukemia worldwide [1]. In patients diagnosed before 60 years of age, it is curable in 35–40% of cases. However, only 5–15% of those presenting with the disease later in life can be cured [2–5]. Almost half of patients reach complete remission, but approximately 10% of patients have a median survival of only 1 year. For more than four decades since the combination of an anthracycline (most often daunorubicin) and cytarabine was first used for standard therapy, the “3 + 7” regimen has remained the standard therapy for AML [6]. The long term disease-free survival of AML patients under age 60 remains around 40%, with minimal improvement over the past several decades. The chemotherapy effectiveness may have hit a ceiling for treating AML, especially for older patients and those who either tend to relapse or have intermediate- or high-risk factors associated with AML [7]. FMS-like tyrosine kinase 3 internal tandem duplication (FLT3-ITD) mutations is over-expressed in 20–30% of AML cases and the most common molecular alteration in AML [8]. Patients with FLT3-ITD-mutated acute myeloid leukaemia, particularly those with a high allelic frequency, relapse quickly and have a shortened overall survival compared with patients who have the wild-type FLT3 [9]. It is well known that it is notoriously hard to treat [10]. Thus, an novel effective therapeutic drugs for the patient of AML, especially FLT3-ITD mutation are vital.

Berberine (BBR), a quaternary proto-berberine isoquinoline alkaloid, is a well-known naturally occurring medicine derived from the root and the stem bark of numerous plants such as *Hydrastis canadensis*, *Berberis aquifolium*, *Berberis aristata*, *Berberis vulgaris* and many other plants [11]. BBR is widely used for gastrointestinal infections and various inflammations in Asian countries [12]. It has many pharmacological actions, including activity against tumors [13, 14], microbe [15], inflammation [15, 16], neurodegenerative disease [17, 18] and other diseases. Recent studies have found that BBR exerts cytotoxicity and inhibits telomerase and topoisomerase, promotes the proliferation apoptosis in human leukemia cells by specifically binding to oligonucleotides or polymorphic nucleic acid and by stabilizing DNA triplexes or G-quadruplexes; the electrostatic interactions may be quantified in terms of the Hill model of cooperative interactions [19–22]. BBR showed antiproliferative activity and promptly localized to the nucleus 5 min to 15 min after BBR treatment in HL-60 human promyelocytic leukemia cells [22]. And also, research reported that BBR significantly inhibited the viability of AML THP-1 cells without incurring cytotoxicity to normal monocytes, which suggests that BBR could be an effective therapeutic agent against AML leukemia cells [23].

Importantly, we recently found that BBR effectively inhibits MV4-11 cells, an FMS-like tyrosine kinase 3 (FLT3)-mutated human AML cell line that is resistant to the commonly used anti-leukemia drug cytarabine (Ara-C) [24]. Based on these data, BBR may provide a new strategy for AML treatment, especially FLT3-mutated AML. However, BBR has extremely poor oral bioavailability (< 1%) [25], because of its poor aqueous solubility, extensive intestinal elimination and hepatobiliary excretion, as well as the Pgp-mediated efflux and the multidrug efflux pump induced low intestinal permeability [25–29]. Therefore, it is very important to design a formulation that improves the water solubility and facilitates the oral bioavailability of BBR.

Self-nanoemulsifying systems serve as a means to improve the bio-safety of lipophilic compounds and to improve their solubility, membrane transport, and absorption via the lymphatic system by bypassing first-pass metabolism [30]. A higher solubilization capacity, rapid onset of action, and reduced inter-subject differences are the advantages of self-nanoemulsifying system over with conventional systems [31]. In recent years, this system has emerged as a promising new technique to increase solubility, reduce side effects and enhance anti-tumor capacity [32]. However, there has been no study concerning self-nanoemulsifying systems encapsulating BBR for treatment of relapsed/refractory AML.

In this study, a novel self-nanoemulsifying system of BBR was prepared using a low-energy emulsification method to greatly improve BBR solubility. Additionally, we studied the in vitro release and in vivo pharmacokinetic characteristics of this system and investigated the mechanism by which it increases BBR oral bioavailability by using a Caco-2 cell monolayer model. Importantly, the anti-leukemia activity of the system was evaluated in an MV4-11 xenograft mouse model.

## Methods

### Materials

MV4-11 and Jurkat cells were obtained from American Type Culture Collection (ATCC, Manassas, USA) and cultured in IMDM (HyClone, Thermo scientific, USA) at 37 °C with 5% CO_2_. Caco-2 cells were obtained from the Biomedical Analysis Center, TMMU and were cultured in DMEM (HyClone, Thermo scientific, USA) at 37 °C with 5% CO_2_. All cells were frozen in liquid nitrogen in 90% FBS (HyClone, Thermo scientific, USA) and 10% DMSO at − 196 °C. Cells of passages 5–10 were used in the study.

### In vitro anti-acute myeloid leukemia activity assay

The anti-AML activity of BBR against Jurkat and MV4-11 cells was studied using MTT assays. Cells were seeded at 1 × 10^5^/mL in 96-well plates exposed to BBR suspension (Baoja Natural Plant Development Co. Ltd. Baoji, Shan’xi, China) at various concentrations (10, 20, 50, 100, 150, 200 µg/mL) and 200 μg/mL cytosine arabinoside water solution (Ara-C, Pfizer, NY, USA) incubated after treatment for 24 h. Absorption at 450 nm was measured using an iMark Microplate reader (Bio-Rad, USA) after added 10 μL/cell of CCK-8. Cell viability was calculated: Cell viability (%) = (OD_treatment_/OD_control_) × 100%. Each experiment was performed in triplicate.

### System component determination

The solubility of BBR in the oils (peanut, saxol and squalene), surfactants (Tween 80, Cremophor EL40, Cremophor RH40) and co-surfactants (ethanol, glycerin and propylene glycol), was investigated by adding excess BBR to these compounds. Then, the samples were mixed for 60 s and shaken for 24 h in a water bath at 37 °C to reach equilibrium. Finally, the concentration of BBR in these mixtures was determined using high-performance liquid chromatography (HPLC, Agilent 1260; Symmetry C18 column [5 μm, 4.6 × 250 mm]; flow phase: acetonitrile/0.03 M potassium dihydrogen phosphate solution = 40/60; flow rate: 1.0 mL/min; detection wavelength: 263 nm; injection volume: 10 μL).

### Optimize drug additive determination of this system

Based on a preliminary experiment, the system was composed of squalene (oil), RH40 (surfactant), and 1,2-propanediol (co-surfactant). The Smix surfactant/co-surfactant ratios were 3:1, 4:1 and 5:1. A series of Smix and oil ratios (1:9, 2:8, 3:7, 4:6, 5:5, 6:4, 7:3, 8:2 and 9:1) were added and then gently agitated until the samples had a transparent appearance. The physical state of the self-nanoemulsifying system is shown in a pseudo-phase diagram in which one axis represents the water, another axis represents the oil and a third axis represents the mixture of co-surfactant and surfactant; the diagram was prepared using Origin 7.0 software (Origin Lab Corporation, USA) [33, 34]. To determine the optimum BBR addition amount, four concentrations (0.1%, 0.2%, 0.5%, and 1%) were examined. These samples were prepared using previously described methods.

### Preparation of the self-nanoemulsifying system

In general, Smix was prepared by mixing RH 40 and 1,2-propanediol at a 4:1 (w/w) ratio. Next, squalene was added to Smix ratio of 8:2 (Smix: squalene) and berberine. The mixture was added drop-wise under gentle agitation to the water phase. In addition, the mixtures were assessed in this system when they were at low viscosity and showed a clear appearance. Blank control (blank SNE) was obtained as described previously, except the drug was replaced with water.

### Morphological and physicochemical characteristics

The ultra-structure of this system was observed using previously described methods [35]. All samples after dilution 50 times with water were examined with a JEM-1230 transmission electron microscope (TEM, JEOL Ltd., Tokyo, Japan) at 120 kV voltage after negative straining with 1% phosphotungstic acid solution (pH 7.4). The morphology of the samples was subsequently characterized by JEM-6700F scanning electron microscopy (SEM, JEOL Ltd., Tokyo, Japan). Physicochemical characteristics, including size, polydispersity index, and zeta potential were determined with a Nano ZS 90 (Malvern Instruments Ltd., UK).

### Encapsulation efficiency and drug loading

Encapsulation efficiency (EE) and drug loading (DL) of BBR SNE were investigated based on previously reported methods [33]. Briefly, 0.2 mL of blank SNE or BBR SNE was added to 0.4 mL of absolute ethanol. Then, the samples were centrifuged (13,000×*g*, 30 min) after mixing and shaking for 60 s to ensure complete emulsification. The precipitates were added to 0.3 mL of water after the supernatants were removed. The BBR content of the precipitates and supernatants was determined via HPLC. The following equations were used to calculate encapsulation efficiency (EE) and drug loading (DL): EE (%) = (Actual/Theoretical drug loading) × 100%. DD = Actual drug loading/sample volume.

### Physical state and drug interaction evaluation of this system

The physical state and drug interaction of the system was evaluated via differential scanning calorimetry (DSC) and thermal gravimetric (TG) analysis based on the previously method [36]. The samples were examined using a Q600 instrument (TA Instruments, New Castle, USA) at a heating rate of 10 °C/min from room temperature up to 200 °C under a nitrogen atmosphere. The weight loss of BBR and BBR SNE were measured, and samples were carefully sealed in an aluminum plate. The FTIR absorption data of the suspension (BBR) and self-nanoemulsion delivery system (BBR SNE) samples after preparation using the KBr disk method were obtained using a Lambda 950 spectrometer (PerkinElmer, Boston, USA) with a resolution of 4 cm^−1^ at 64 scans in the range of 400–4000 cm^−1^. By monitoring the locations of bands and peak frequencies, all interactions and chemical changes were determined.

### Stability and release profile study of this system

#### Stability study

The stability study was adapted from previously described methods [37]. The stability of the system was evaluated by centrifugation (13,000 rpm for 30 min). In addition, the appearance of the samples was observed after six cycles (one cycle stored at 4 °C and another at 25 °C, both for 48 h). The system was considered unstable if it showed various appearances, such as creaming, drug or phase separation, turbidity, de-emulsification or precipitation.

#### In vitro release profile in PBS

BBR SNE and BBR were studied using the dialysis bag diffusion technique in vitro. Briefly, 5-mL samples (5 mg/mL) were added to a dialysis bag (SP132574, MWCO 10,000 g/mol, Sangon Biotech, Shanghai, China) separately. Then, the samples were immersed in 200 mL of PBS (0.1 M, pH = 7.4) release media and mixed at 100 rpm. The concentrations of the samples were measured by HPLC after release at various times (0.17, 0.33, 0.5, 1, 1.5, 2, 4, 6, 9, 12, 24, 36 and 48 h) with previously describe detection condition. Each experiment was performed in triplicate.

### In vivo pharmacokinetic studies

Rabbits were obtained from the Second Xiangya Hospital of Central South University (Hunan, China) and were randomly divided into two groups (BBR and BBR SNE, n = 6). Rabbit blood (0.5 mL) was collected via the contra lateral vena cava auricular vein at 0, 0.17, 0.33, 0.5, 0.75, 1, 2, 4, 8, 12, 18 and 24 h after oral BBR equivalent administration at 50 mg/kg, based on the previously reported dose [38]. Blood samples were immediately separated by centrifugation at 4000 rpm for 10 min and stored at – 20 °C. All samples were analyzed via HPLC. Pharmacokinetic parameters were estimated using the noncompartmental methods by drug and statistic (DAS) software.

### Caco-2 cell cytotoxicity and transport studies

#### Caco-2 cell cytotoxicity

Caco-2 cells were seeded in 96-well plates at a density of 1 × 10^4^ cells/well and cultured in an incubator at 37 °C under 5% CO_2_. The cell medium was replaced with the suspension and the BBR system at a series of finally concentrations (6.25, 12.5, 25, 50, 100, 500 μg/mL), and the cells were incubated for 24 h. The Caco-2 cell cytotoxicity of the samples was determined using a Bio 6.0 Microplate Reader at 490 nm via the same MTT method. Each experiment was performed in triplicate.

#### Caco-2 cell monolayer model transport studies

Cells (1.5 × 10^5^ cells/well) were added in 24-well Transwell plates (0.4 µm, Corning, USA) at 37 °C in a 5% CO_2_ humidified atmosphere. The culture medium was replaced every 2 days for the 1st week and every day thereafter. Cells were cultured for 16–21 days prior to the following cellular uptake experiments. The integrity of the cell monolayers was monitored by measuring transepithelial electrical resistance (TEER). Only when the TEER value was > 300 Ω cm^2^ were the cell monolayers used in subsequent studies.

We used 50 μg/mL verapamil and 25 μg/mL BBR or BBR SNE in HBSS. The experiment was performed in both the apical to basolateral (AP–BL) and basolateral to apical (BL–AP) direction. Briefly, 200 μL of drug with or without verapamil was added to the AP side in the AP–BL experiments, and 800 μL HBSS was added to the BL side. For the BL–AP experiments, 800 μL of drug solution with or without verapamil was added to the BL side, and 200 μL of blank HBSS was added to the AP side of the monolayer as the acceptor phase. Then, 50 μL of the samples were taken from the AP side at 15, 30, 60, 90, 120 and 180 min time points and equal volumes of blank HBSS were added after each withdrawal. The samples were immediately stored at − 80 °C for subsequent analysis. Finally, samples were analyzed via HPLC. The apparent permeability coefficient (Papp) and efflux ratio (ER) were determined using the following equations: Papp = dQ/dt/(A∙C0); ER = Papp (BL–AP)/Papp (AP–BL), where dQ/dt is the rate of permeability, A is the surface area of the filter membrane, and C0 is the initial concentration of insulin in the apical chamber. Each experiment was performed in triplicate.

### Anti-tumor activity against the MV4-11 xenograft model in vivo

Animal maintenance and experimental procedures were carried out in accordance with the National Institutes of Health Guidelines for the Use of Experimental Animals. The MV4-11 xenograft mouse model was prepared according to previously described methods [39, 40]. Briefly, Spleen cells (5 × 10^6^ cells/mouse) from MV4-11 transplanted NSG mice were intravenously injected into NSG (female, 6 weeks, Beijing Vitalstar Biotechnology Co., Ltd) mice via tail vein. One week after leukemia cell injection, all mice were randomly divided into five groups (PBS control, Blank SNE control, BBR, Ara-C, BBR SNE, n = 10). All animals were treated at the same dose of 50 (mg/kg)/day for 14 days by oral administrated with PBS, Blank SNE, BBR, and BBR SNE except Ara-C was administrated by tail vein injection, referring to the effect dose of Ara-C in the MV4-11 model [41]. During 98 days of observation, the survival time and number of all mice were recorded.

### Statistical analysis

All the data are expressed as the average ± SD (standard deviation). Differences between two groups were tested using Student’s *t*-test. Differences in all data were analyzed using Student’s *t*-test with GraphPad Prism 5.0 software (San Diego, USA). Differences are expressed as ***P < 0.001, **P <* 0.01* or *P <* 0.05.*

## Results and discussion

### Berberine exerted anti-acute myeloid leukemia activity in vitro

**Fig. 1:**
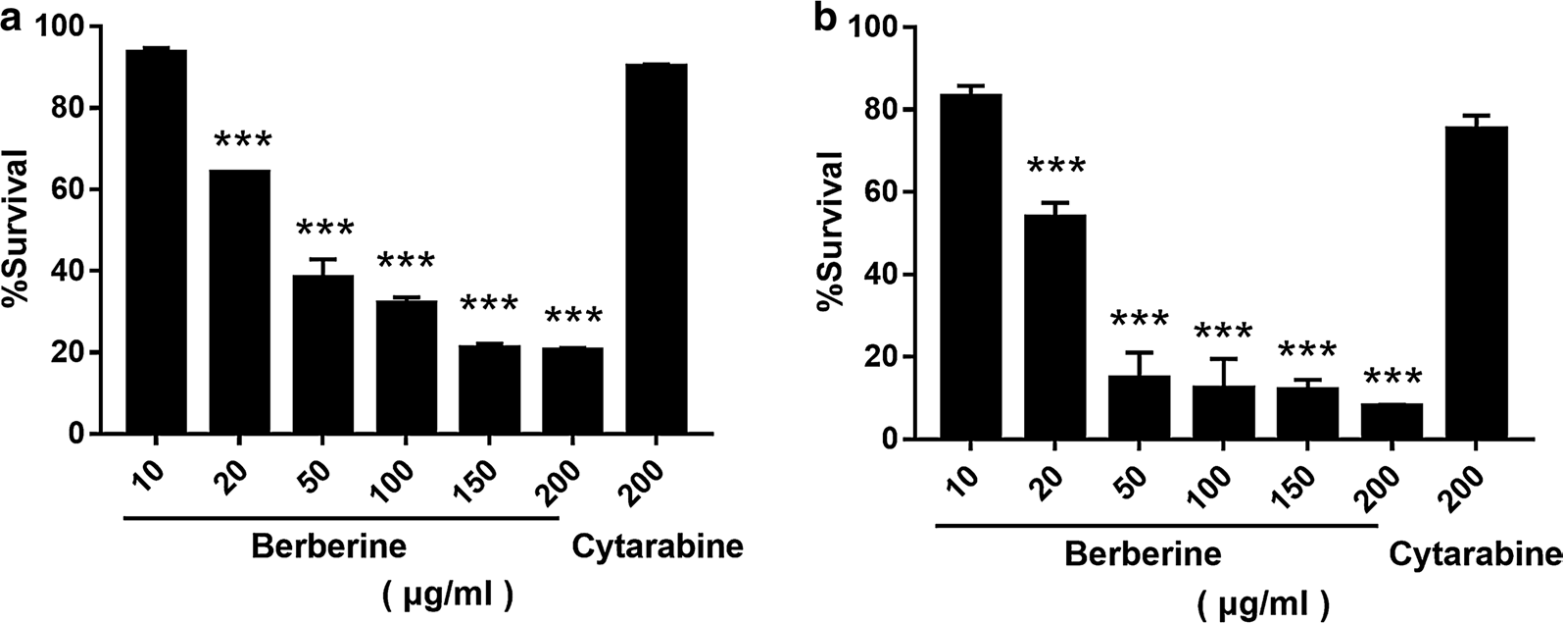
Effect of a series of berberine and cytarabine solutions on the MV4-11 cell line at **a** 24 h and **b** 48 h. The data are expressed as the mean ± S.D. (n = 3); ***P < 0.001, **P < 0.01, and *P < 0.01. Vs: compare with 10 μg/mL berberine

To investigate the in vitro anti-AML activity of BBR, an MTT assay was used, and the cytotoxicity was measured 24 h (Fig. 1a) and 48 h (Fig. 1b) after treatment. Figure 1a, b shows the inhibition activity of BBR in MV4-11 cells. Cytarabine, a commonly used clinical anti-AML drug, was used as control. At a concentration of 10 μg/mL, BBR showed no obvious cytotoxicity toward MV4-11 cells at 24 or 48 h after treatment. Similarly, 200 μg/mL of cytarabine failed to decrease the viability of MV4-11 cells. However, when the concentration was higher than 20 μg/mL, BBR significantly induced cell death in a dose-dependent manner. We also performed the same test in Jurkat and HL-60 cells, obtaining similar results (Additional file 1: Figures S1A, S1B, S2A and S2B), with the only difference being that 200 μg/mL of cytarabine induced cell death in Jurkat and HL-60. Thus, the activity was approximately equal to 20 μg/mL BBR but was significantly weaker than that of 50 μg/mL BBR.

### Component determination of the system

The solubility of BBR in Tween-80, RH40 and EL40 was 0.43 ± 0.13 mg/g, 1.67 ± 0.22 mg/g, and 1.35 ± 0.31 mg/g, respectively. Additionally, the BBR solubility in the three co-surfactants 1,2-propanediol, glycerin and ethanol was 3.78 ± 0.32 mg/g, 1.23 ± 0.45 mg/g, and 1.55 ± 0.23 mg/g, respectively. Furthermore, in the three oils (peanut, saxol and squalene), the solubility was 0.37 ± 0.21 mg/g, 0.43 ± 0.12 mg/g and 2.13 ± 0.55 mg/g, respectively. Based on these solubility results, this nanoemulsifying system was prepared with RH-40, 1,2-propanediol, and squalene.

### Formation screening of the system

**Fig. 2:**
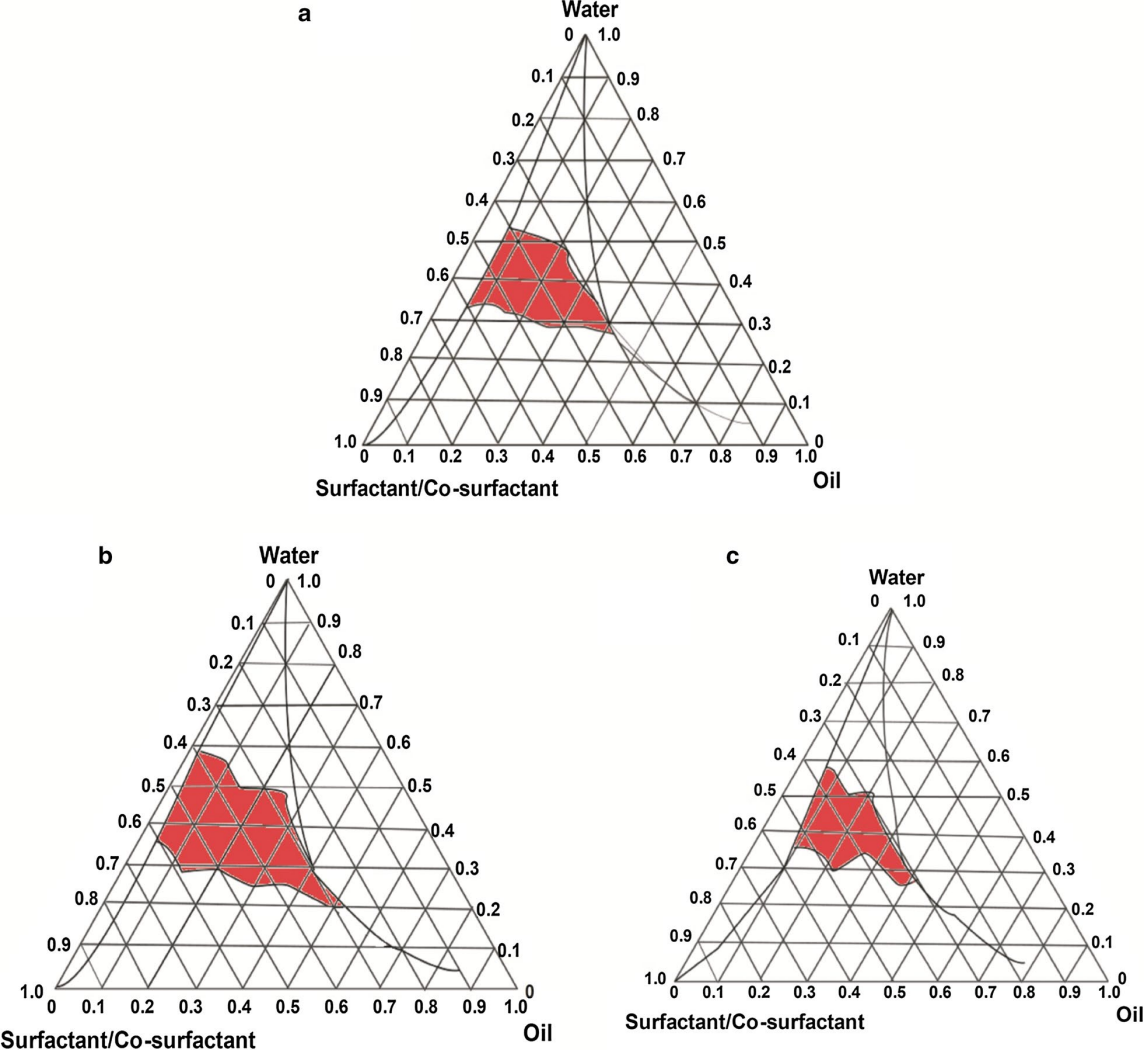
Pseudo-ternary phase diagrams of the self-nanoemulsifying system composed of the following constituents: RH-40 (surfactant), 1,2-propanediol (co-surfactant), squalene (oil phase). **a** Surfactant and co/surfactant mixture ratio (Smix), 3:1; **b** Smix, 4:1; **c** Smix, 5:1. The red areas represent the nanoemulsion formation regions

Pseudo-ternary phase diagrams were prepared separately for various Smix ratios. The nanoemulsion areas were initially enlarged and then reduced with an increase in Smix (Fig. 2a–c). Additionally, the formation nanoemulsion area of Smix 4:1 (Fig. 2b) was demonstrated to be larger than that of the other Smix ratios (3:1, Fig. 2a; 5:1, Fig. 2c), which showed small and narrow areas. According to these pseudo-ternary phase diagrams, an Smix of RH-40/1,2-propanediol = 4:1, with the largest area, was the best formulation for the self-nanoemulsifying system.

**Fig. 3:**
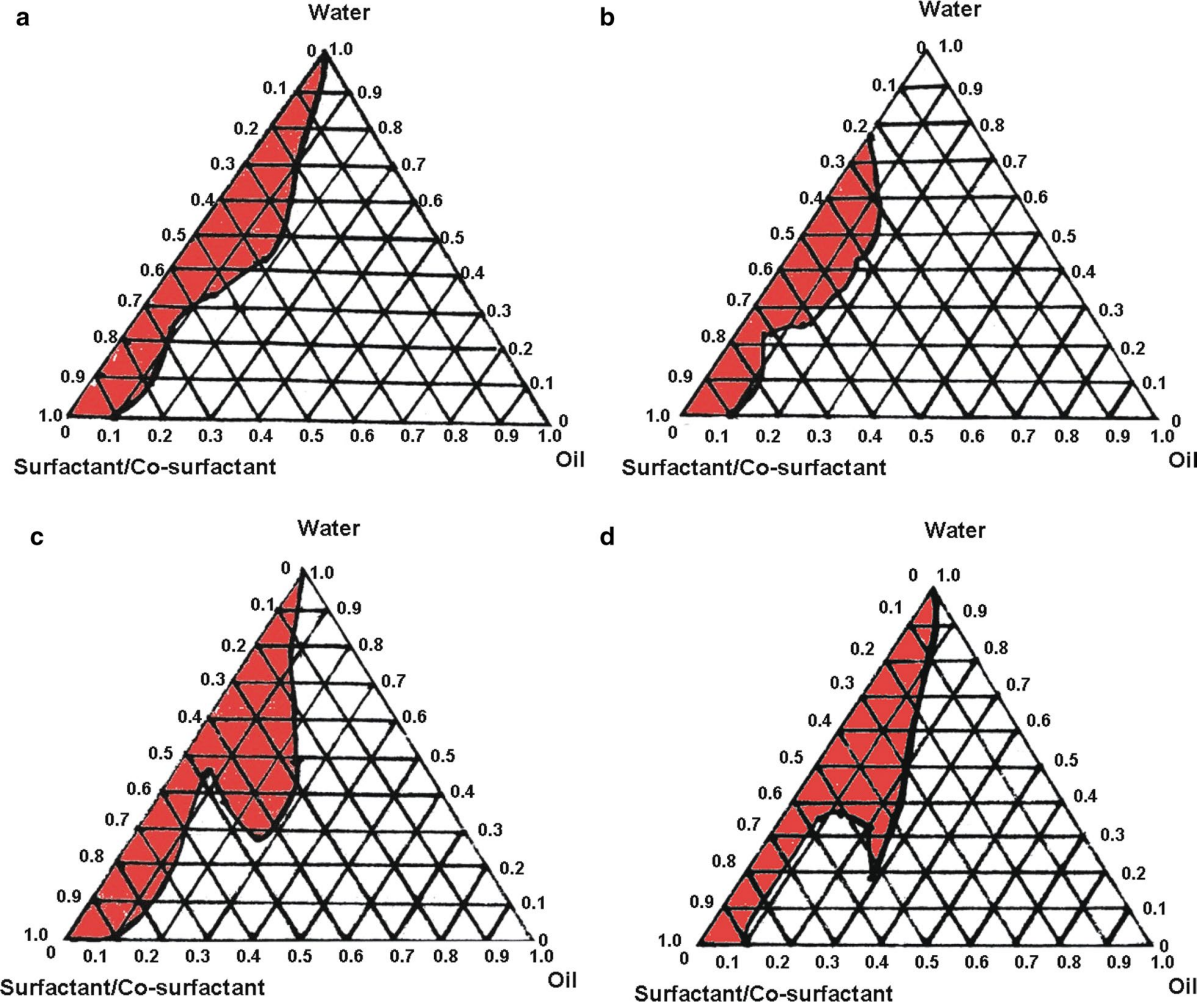
Pseudo-ternary phase diagrams of the system (Smix = 4:1) containing different berberine concentrations. **a** 0.1%; **b** 0.2%; **c** 0.5% and **d** 1.0% (w/w). The red areas represent the nanoemulsion formation regions

Depending on the system loading with BBR, drug content could affect the nanoemulsion formation area focus. These areas first enlarged and then reduced with an increase in drug content (Fig. 3a–d). Additionally, the nanoemulsion formation area of 0.5% BBR SNE (Fig. 3c) was larger than that of the other contents (0.1, Fig. 3a; 0.2%, Fig. 3b and 1%, Fig. 3d), all of which showed small and narrow nanoemulsion areas. Overall, a significant reduction in self-nanoemulsion area was evident only at 1% (Fig. 3d). Additionally, these results demonstrated that producing this system with high drug content (> 0.5%) does not confer an added stability benefit, a beneficial finding for industrial manufacture of self-nanoemulsifying products. Therefore, we identified the optimal drug content to be 0.5%.

### Preparation and characterization of this system

**Fig. 4:**
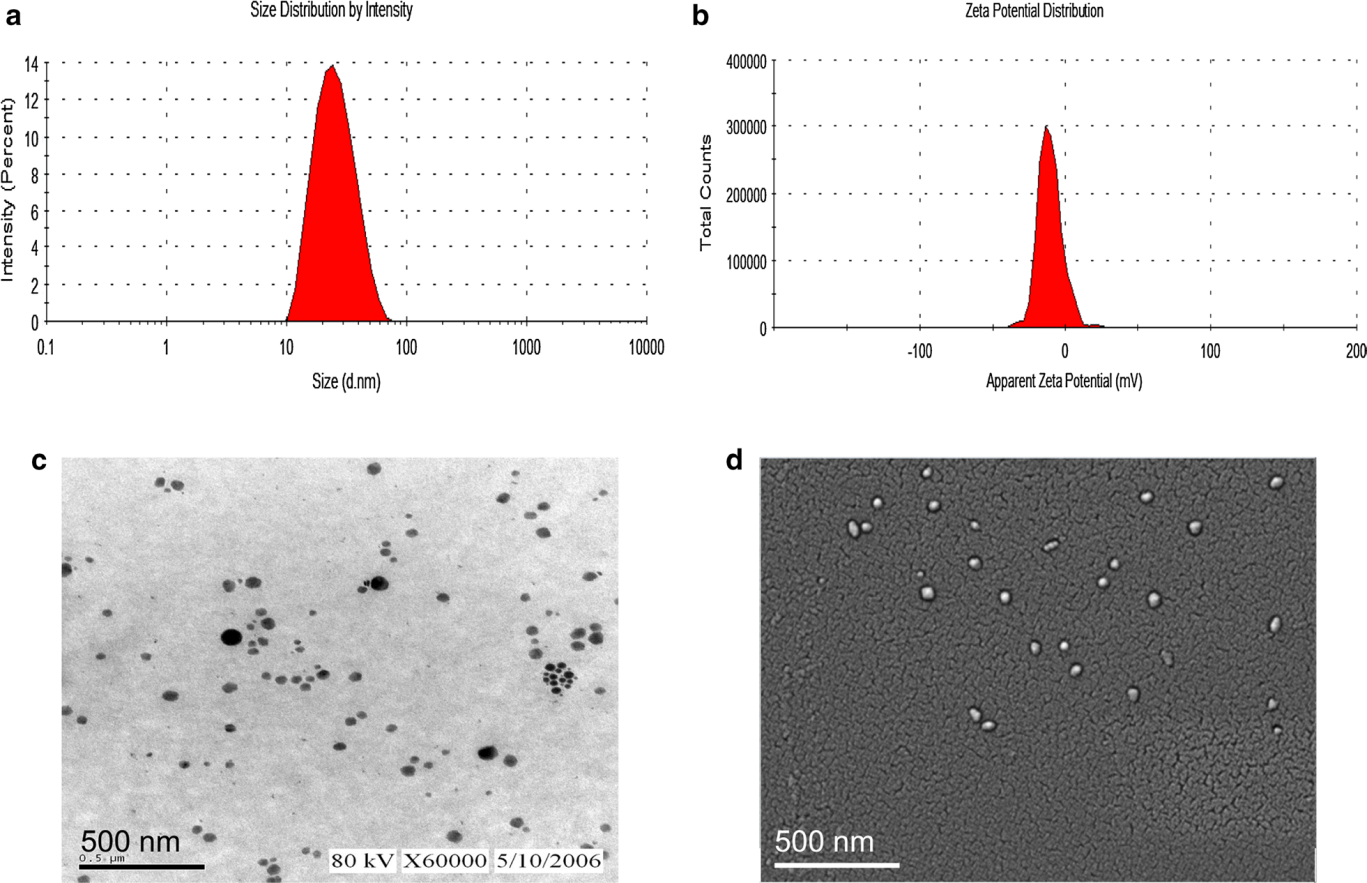
Characteristics of the self-nanoemulsifying system. **a** Particle-size distribution; **b** zeta-potential distribution; **c** transmission electron micrograph and **d** scanning electron micrograph

Based on the solubility and pseudo-ternary phase diagram results, a self-nanoemulsifying system (BBR SNE) with 0.5% BBR, 9.6% RH-40, 2.4% 1,2-propanediol, and 0.3% squalene was prepared using the phase inversion method. The BBR suspension was a suspended, turbid solution, while the novel system was a clarified, transparent solution. In this study, we prepared BBR SNE with 2.5-fold improvement in water solubility compared with that of BBR (2 mg/mL). Figure 4a shows that the mean size of these system particles was 23.50 ± 1.67 nm; 75% of the particles were smaller than 28.46 nm, and 90% of the particles were smaller than 37.24 nm. The PdI value was directly proportional to the particle size range. In this study, the PdI was 0.121 ± 0.01, which is less than 0.3. This result suggests that the novel self-nanoemulsion delivery had a relatively limited particle-size distribution. As shown in Fig. 4b, the zeta potential was 4.17 ± 0.82 mV. We next observed the morphology of this novel system using TEM (Fig. 4c) and SEM (Fig. 4d). Figure 4c, d shows that most of the droplet particles were between 1 and 100 nm, and the system was spherical when observed under both TEM and SEM. These results showed that this system had good characteristics and could fulfill the formation requirements. We detected the loading efficacy and drug loading amount of BBR SNE. We found that the loading efficacy and drug loading amount were 82.5 ± 2.13% and 4321.23 ± 3.33 µg/mL, respectively.

### Evaluation of the physical state and drug interaction

**Fig. 5:**
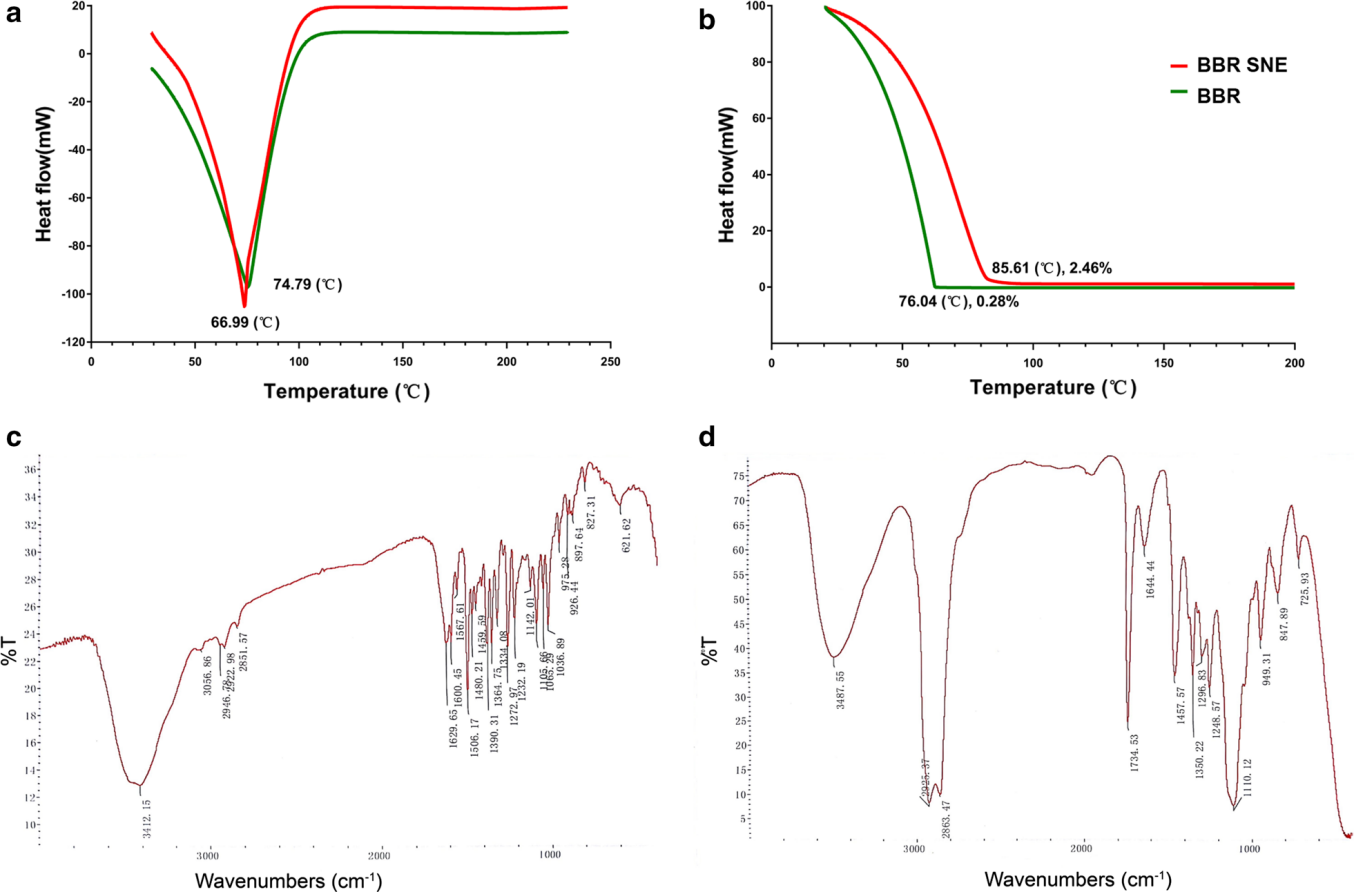
Evaluation of the physical state and drug interaction of this system. **a** Differential scanning calorimetry; **b** thermogravimetric analysis; **c** FTIR spectrum of the berberine suspension; **d** FTIR spectrum of the system

A characteristic single prominent peak was observed at 66.99 °C in the thermogram of the nanoemulsifying system that was absent in the thermogram of the BBR suspension (Fig. 5a). The DSC thermogram of the BBR suspension showed one peak at 74.79 °C. We observed that this system lowered the melting point of the drug, suggesting a possible intracellular permeation mechanism in this system. One of the simplest interactions is hydrogen bonding between the drug chains, invoking hydrogen-bonded beta-structure interactions between the drug and system.

The temperature exhibiting the highest rate of weight loss was indicated by a peak in the thermogravimetric analysis data. The self-nanoemulsifying system and BBR suspension showed similar weight loss patterns with just one step (Fig. 5b). The weight loss of this delivery began and ended at approximately 30 and 85.61 °C, respectively. The weight loss of the BBR suspension began and ended at approximately 30 and 76.04 °C, respectively. The thermogravimetric curve of the BBR suspension showed a weight loss of 0.28% within a specified temperature from 0 to 200 °C due to the evaporation of the water content of the self-nanoemulsion delivery system. Another decrease of 2.46% in the mass profile of this system occurred over a temperature range from 0 to 200 °C. Mass transfer from a consistent viscous liquid into the gas state owing to the evaporation process is typically more difficult than that from a less viscous liquid.

FTIR spectra of the water solution and its system (Fig. 5d) indicated sharp peaks with proper intensities; vibration changes play a significant role in the intermolecular interactions in this system. In the FTIR spectra, the characteristic peaks were as follows: 700–1300 cm^−1^ (skeletal C–C vibrations), 1105.66 cm^−1^ (C–O), 1600.45 cm^−1^, and 1506.17 cm^−1^ aromatic C=C stretching and skeleton vibration of aromatic C=C ring stretching, 1390.31 cm^−1^ and 1364.75 cm^−1^ (C=C stretching), 1272.97 cm^−1^ (C–O–C stretching), and 1035.89–1142.01 cm^−1^ (in plane =C–H bending). A characteristic carbonyl functional group peak at 1644 cm^−1^ along with the characteristic C–O–C stretching vibrations of the repeated –OCH2CH2 chain of TPGS were observed in the region of 1104–1268 cm^−1^ (Fig. 5d). The absence of the characteristic peak of BBR at 1734 cm^−1^ confirmed entrapment in this system (Fig. 5d). These data showed that this system successfully loaded BBR.

### Stability and release profile study of the delivery system

This novel system showed good thermodynamic and high centrifuge stability because it exhibited no creaming, de-emulsification, drug separation, turbidity precipitation, and/or phase separation after centrifugation at 13,000 rpm for 30 min. These results demonstrated that the delivery system had good thermodynamic stability.

**Fig. 6:**
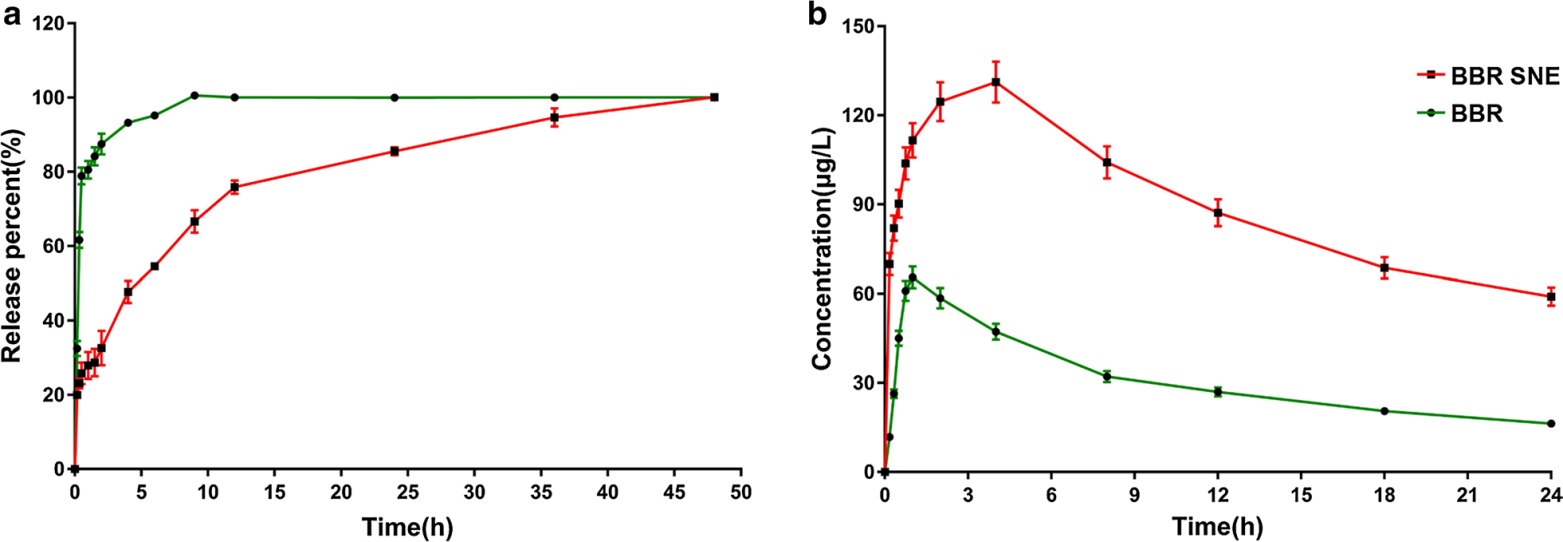
In vitro release and in vivo pharmacokinetic studies. **a** In vitro release profile of the berberine suspension (BBR) and the system (BBR SNE) in PBS (pH = 7.4) (n = 3); **b** plasma concentration profiles of berberine after oral administration of BBR and BBR SNE in healthy rabbits (n = 6)

Release studies of the system and water solution were performed in PBS (pH = 7.4). As shown in Fig. 6a, approximately 90% of BBR was released from suspension in 2.5 h, while 90% of the drug was released from the system in 36 h. Additionally, the near complete release (90% release rate) time of the system was delayed 14.4-fold compared with that of the suspension. A possible reason for the delayed drug release from the system is that the drug was perhaps adsorbed on the surface and dissolved.

### In vivo pharmacokinetic studies

The pharmacokinetic parameters of this novel system and the BBR suspension were compared. Figure 6b shows the plasma concentration profiles of BBR after oral administration of both formulations in rabbits. The pharmacokinetic parameters are summarized in Table 1. The AUC of the system (3639.94 ± 10.37 µg/mL h) was 3.41-fold greater than that of the BBR suspension (1071.26 ± 6.15 µg/mL h), indicating 341% relative bioavailability of the system compared with the suspension formulation. The MRT was also significantly prolonged (1.52-fold) for the system (3.85 ± 0.11 h) compared with the suspension (2.54 ± 0.12 h). These results suggested that the system promoted absorption and sustained release of BBR, thereby improving the relative bioavailability of BBR.

**Table 1:** The pharmacokinetics parameters of berberine nanoemulsion in healthy rabbits

Parameters	Unit	Berberine suspension	Berberine nanoeumlsion
AUC_0–24_	µg/mL h	1071.258 ± 6.147	3639.937 ± 10.367**
AUC_0–∞_	µg/mL h	1263.456 ± 1.472	4319.261 ± 26.784***
MRT_0–24_	h	12.195 ± 2.581	23.256 ± 1.768***
MRT_0–∞_	h	16.722 ± 1.732	42.467 ± 2.493***
Ka	1/h	2.581 ± 0.214	0.710 ± 0.369
T_max_	h	1.379 ± 0.247	4.205 ± 0.234
C_max_	µg/mL	62.466 ± 0.248	113.699 ± 0.369**
t_1/2_α	h	1.065 ± 0.231	2.001 ± 0.278**
t_1/2_β	h	16.649 ± 0.321	21.241 ± 2.296**
t_1/2_Ka	h	0.269 ± 0.026	0.976 ± 0.325

Values are the mean ± SD, n = 6

*AUC* Areas under the curve, *MRT* mean retention time, *T*_*max*_ peak time, *C*_*max*_ pea concentration, *t*_*1/2*_*α* half-life of distribution, *t*_*1/2*_*β* half-life of elimination, *t*_*1/2*_*Ka* half-life of absorbance

** P < 0.01, *** P < 0.001, compared with berberine suspension

### Caco-2 cell cytotoxicity

**Fig. 7:**
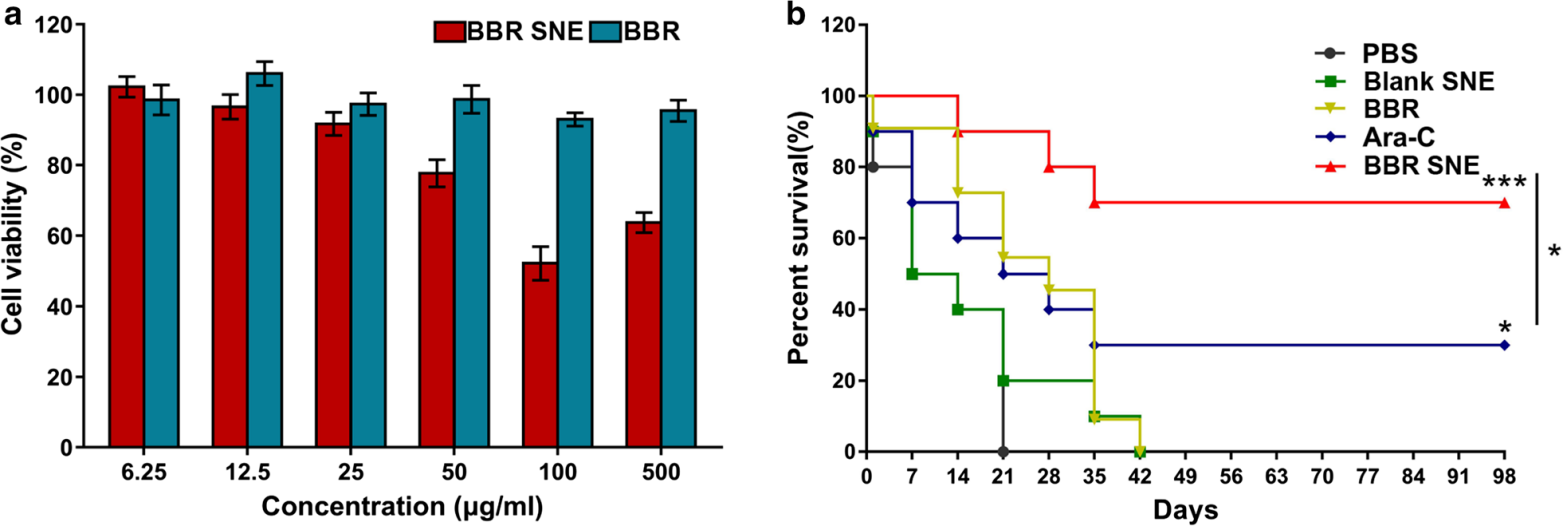
Effect of cell viability and survival of the self-nanoemulsifying system. **a** Effect of berberine solution (BBR) and the system (BBR SNE) on Caco-2 cell viability as evaluated by MTT assays after treatment for 24 h. The data are expressed as the mean ± S.D. (n = 3). **b** The survival rate of MV4-11 xenograft mice orally administered berberine suspension (BBR), PBS, Blank SNE and the system (BBR SNE) excepted for cytarabine (Ara-C) with the vein intravenous injection at 50 (mg/kg)/day for 14 consecutive days; the same volume of PBS and blank SNE were used as controls. The data are expressed as the mean ± S.D. (n = 10); *P < 0.05, and ***P < 0.001

A CCK-8 assay was conducted to assess the cytotoxicity of the system and BBR solution (Fig. 7a). The BBR SNE showed no significant cytotoxicity (cell viability > 90%) at concentrations between 6.25 and 500 μg/mL, while BBR showed no significant cytotoxicity at concentrations between 6.25 and 25 μg/mL. Thus, a concentration of 25 μg/mL BBR and BBR SNE was selected for Caco-2 cell monolayer transport studies.

### Caco-2 cell monolayer transport studies

Experiments were performed in the apical to basolateral (AP–BL) and basolateral to apical (BL–AP) direction. As shown in Table 2, for the Caco-2 cell monolayer transport study (AP–BL), the absorptive concentration–time profiles of the BBR suspension were too low to be detectable by HPLC. The efflux ratio of BBR SNE was 2.47 ± 0.47, suggesting the presence of an efflux transporter. In addition, the apparent permeability coefficient (Papp) values of BBR from A-to-B transport and from B-to-A efflux were summarized [27]. The permeability coefficients of BBR and BBR SNE with verapamil, a P-glycoprotein inhibitor, were significantly lower than with BBR, which indicated that BBR SNE reduced BBR efflux in Caco-2 cell monolayers, and the drug was probably effluxed by P-glycoprotein.

**Table 2:** Apparent permeability coefficients (Papp) and efflux ratio (ER) of berberine and berberine SNE cross Caco-2 monolayer

Group	Papp (×10^−8^ cm/s)		ER
	AP–BL	BL–AP	
Berberine	–	2.771 ± 0.213	–
Berberine SNE	0.749 ± 0.116	1.852 ± 0.271**	2.473 ± 0.467
Berberine SNE + Ver	0.232 ± 0.231*	1.325 ± 0.172^#^	5.711 ± 0.524^##^

Values are the mean ± SD, n = 3

*ER* efflux ratio, *Papp* permeability coefficient, *SNE* self-nanoemulsifying system, *Ver* verapamil

* P < 0.05, compared with berberine NE in AP–BL transport

** P < 0.01, compared with berberine in BL–AP transport

^#^P < 0.01, compared with berberine NE in BL–AP transport

^##^P < 0.01, compared with berberine NE

### Anti-tumor activity against an MV4-11 xenograft model in vivo

We evaluated the therapeutic performance of BBR SNE using the MV4-11 xenograft model. Mice were treated with the system and/or the suspension (50 mg/kg). Mice received PBS, blank SNE and cytarabine (Ara-C) as controls. Mice were orally administered the corresponding material within an experimental period of 14 days (Fig. 7b). The mice treated with the system or with Ara-C displayed higher survival rates (70/30% at 98 days, respectively) than those treated with BBR, PBS and blank SNE (no survival). These data suggested that treatment with this novel system markedly increased the survival rate of MV4-11 xenograft mice.

## Discussion

In high income countries, over 90% of pediatric acute myeloid leukemia (AML) patients achieve complete remission with current intensive chemotherapy protocols. However, even with optimal therapies, 30–40% of patients relapses and faces a dismal prognosis [42]. There are many research showed that FMS-like tyrosine kinase 3 (FLT3) internal tandem duplication (ITD) mutations, resulting in constitutive kinase activity in AML are associated with poor prognosis [43]. The complete response rate was found to be lower and the overall survival shorter than in non-FLT3 AML patients [44, 45]. To date, effective treatment regimens for FLT3 mutant AML patients remain lacking and represent an urgent need [8]. MV4-11 cell line possessing the FLT3 mutation had lower IC50s for azacitidine and panobinostat. In this study, for the first time, we showed that BBR had a strong cytotoxicity effect on the AML cell line MV4-11, which exhibits FLT3 mutations. And also, we had found that BBR significantly inhibit cell death in a dose-dependent manner in the MV4-11, Jurkat and HL-60. Hence, BBR is expected to be a new strategy for treat AML treatment, especially FLT3-mutated AML.

Berberine has been widely used as a broad-spectrum anti-microbial medicine for over 3000 years in China and used for wide clinical applications in Native American and Western medicine [46]. Recent studies have revealed that berberine can trigger autophagic cell death of tumor, and its beneficial effects are receded when the autophagic process is genetically or pharmacologically inactivated, suggesting that autophagy is indispensable for the protective effects of berberine [47]. Research reported berberine induced apoptosis in both HL-60 and WEHI-3 cell lines in association with caspase-3 activity and mitochondria membrane potential depolarization [20]. The clinical use of berberine is largely limited because of its poor intestinal absorption and inadequate plasma level after oral administration [12]. Preclinical studies have shown that BBR has a very limited oral bioavailability (BA) (< 5% in plasma), largely due to its poor aqueous solubility, combined with low gastrointestinal absorption, and rapid metabolism [48]. However, novel self-nanoemulsion system is an emerging area of science and includes material application on the nanometre scale in various domains, including biology, chemistry, medicine, and engineering [49]. It is likely to improve the solubility stability and selectivity of drugs, improving the permeability of drugs while treating solid tumours through the enhanced permeability and retention effect, even for the blood brain barrier, which is high correlated with leukemia metastasis of the bone borrow [50]. There were a few reports focusing on the development of new dosage forms of BBR to increase its bioavailability, such as using the intestinal absorption enhancer, self-microemulsion, and solid lipid nanoparticles [51]. In our study, a novel self-nanoemulsion system was loaded 5 mg/mL berberine with size of 23.50 ± 1.67 nm and zeta potential of − 3.35 ± 0.03 mV was designed using pseudo-ternary phase diagrams to greatly improve BBR solubility. After successful confirmation via TG, DSC, and FTIR analyses, the release profile results showed that this system released BBR more slowly than its solution. And also, the relative oral bioavailability of the system was significantly enhanced by 3.41-fold compared with BBR solution. And also, there is some reported that Berberine-loaded selenium-coated nanostructure lipid carriers (BB-SeNLCs) were more effective and significant in enhancing the bioavailability. The relative oral bioavailability of 663.65%, respectively, compared with berberine solution [12]. Other study showed that the O/W nanoemulsion of BBR showed a relative bioavailability of 440.40% compared with berberine solution. Our data is similar to these describe [27].

It has been reported that delivery of drugs which are P-gp substrates in nanoencapsulation form could be helpful for improvement of bioavailability through circumvention of P-gp efflux system [36]. The Caco-2 cell line is derived from a human colorectal carcinoma. It readily forms monolayers with morphological and functional similarities to the human small intestinal epithelium. In fact the FDA now recognizes the Caco-2 cell monolayer as a viable model that replicates human intestinal absorption [52]. It does this not only because the cells form tight intercellular junctions similar to those of the intestinal epithelium but also because they express ATP-binding cassette (ABC) membrane transporters such as P-glycoprotein (P-gp) and multidrug resistance protein (MRP) that act to protect the body from toxic exogenous substances [53]. In our study, we found that the BA-LB (Papp, ×10^−8^ cm/s) of BBR SNE and BBR was 1.85 ± 0.27 and 2.77 ± 0.21, suggesting the presence of an efflux transporter. This data is also similar in the previous report [27].

The MV4-11 cell inoculated xenograft mouse model has been widely used to evaluate the therapeutic effect of FLT3-ITD-positive AML in vivo [54, 55]. In our study, the mice treated dose with the system or with Ara-C 50 (mg/kg)/day. In the MV4-11 model, the group mice injected with 50 (mg/kg)/day of cytarabine only had reduced tumor growth rates, and the tumor-growth inhibition was 61.94% on the 21st day [41]. And also, there is reported that oral Berberine 200 (mg/kg/day) for 3 weeks statistically significantly reduced the size and weight of spleen in these animals and also reduced the percentage of MAC-3 and CD11b cells in the blood [56]. We consider the dose of pharmacokinetic study of berberine and Ara-C efficacy dose of MV4-11 model. Our data suggest that oral treatment with this novel system significantly prolonged the survival of acute leukemia mice in the MV4-11 engrafted murine model. Of course, more and detail experiments such as other acute leukemia mice model and the mole action mechanism about BBR SNE will be further research. In shortly, in our study, these results demonstrated that a novel system loaded with poorly water-soluble berberine improved the aqueous solubility and oral absorption, and also effectively suppress MV-4-11 tumour growth and drastically increase mice survival rate in vivo.

## Conclusions

We first found that BBR exerted anti-AML activity, especially toward high-risk and relapsed/refractory AML MV4-11 cells in vitro. Additionally, the novel self-nanoemulsifying system with a size of 23.50 ± 1.67 nm and zeta potential of -3.35 ± 0.03 mV was designed using pseudo-ternary phase diagrams to greatly improve BBR solubility. After successful confirmation via TG, DSC, and FTIR analyses, the release profile results showed that this system released BBR more slowly than its solution. The relative oral bioavailability of the system was significantly enhanced by 3.41-fold compared with BBR solution. Furthermore, the Caco-2 cell monolayer transport results showed that the system helped enhance permeation and prevented efflux of BBR. Importantly, we found that oral treatment with this novel system significantly prolonged the survival of acute leukemia mice in the MV4-11 engrafted murine model. These studies confirmed that this novel, efficient system is a promising therapy for acute myeloid leukemia.

## Additional file

**Additional file 1: Figure S1.** Effect of a series of berberine and cytarabine solutions on the Jurkat cell line at (A) 24 h and (B) 48 h. The data are expressed as the mean ± S.D. (n = 3); ***P < 0.001, **P < 0.01, and *P < 0.01. Vs: compare with 10 μg/mL berberine. **Figure S2.** Effect of a series of berberine and cytarabine solutions on the HL-60 cell line at (A) 24 h and (B) 48 h. The data are expressed as the mean ± S.D. (n = 3); ***P < 0.001, **P < 0.01, and *P < 0.01. Vs: compare with 10 μg/mL berberine.
