# Supplementary material for: Self-nanoemulsifying system improves oral absorption and enhances anti-acute myeloid leukemia activity of berberine
Source: J Nanobiotechnology. 2018 Oct 5;16:76. doi: 10.1186/s12951-018-0402-x (PMC6172716; doi:10.1186/s12951-018-0402-x)
Supplement: Supplementary file 1 — Additional file 1: Figure S1. Effect of a series of berberine and cytarabine solutions on the Jurkat cell line at (A) 24 h and (B) 48 h. The data are expressed as the mean ± S.D. (n = 3); ***P < 0.001, **P < 0.01, and *P < 0.01. Vs: compare with 10 μg/mL berberine. Figure S2. Effect of a series of berberine and cytarabine solutions on the HL-60 cell line at (A) 24 h and (B) 48 h. The data are expressed as the mean ± S.D. (n = 3); ***P < 0.001, **P < 0.01, and *P < 0.01. Vs: compare with 10 μg/mL berberine. [file 12951_2018_402_MOESM1_ESM.doc]

**Additional file 1**

# Self-nanoemulsifying system improves oral absorption and enhances anti-acute myeloid leukemia activity of berberine

Jieping Li^1^, Li Yang^1^,Rui Shen^1^, Li Gong^2^, Zhiqiang Tian^3^, Huarong Qiu^4^, Zhe Shi^1^, Lichen Gao^5^, Hongwu Sun^3^*, Guangsen Zhang^6^*

(1.Department of Hematology, Changsha Central Hospital, Changsha, Hunan, 410004, PR China; 2. Department of Clinical Laboratory, the Third Affiliated Hospital, Chongqing Medical University, Chongqing, 401120, P.R. China; 3. Army Military Medical University of Chinese PLA, Chongqing, 400038, PR China; 4. Air Force Military Medical University of Chinese PLA, Xi’an, Shanxi, 710000, PR China; 5.Department of Pharmacy, Cancer Institute, Phase I clinical Trial, Changsha Central Hospital, Changsha, Hunan, 410004, PR China; 6.Department of Hematology, The Second Xiangya Hospital of Central South University, Changsha, Hunan, 410008, PR China)

*Correspondence: Hongwu Sun and Guangsen Zhang

Department of Hematology, The Second Xiangya Hospital of Central South University, Changsha, Hunan, 410008, PR China.

Email: sunhongwu2001@163.com or zgsllzy@163.com


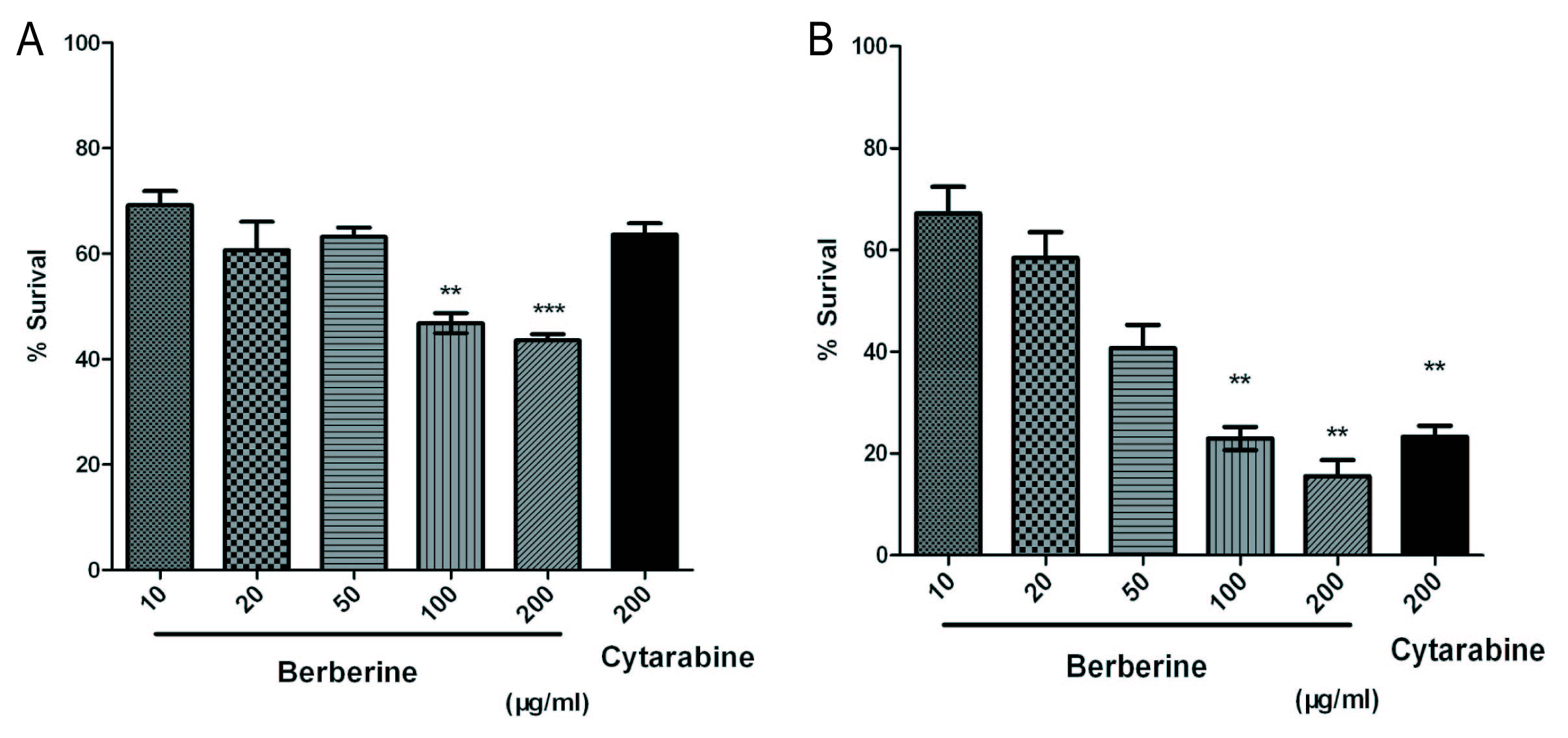


**Figure S1. Effect of a series of berberine and cytarabine solutions on the Jurkat cell line** **at** (A) 24 h and (B) 48 h. The data are expressed as the mean ± S.D. (n = 3); ****P* < 0.001, ***P* < 0.01, and **P* < 0.01. Vs: compare with 10μg/ml berberine.


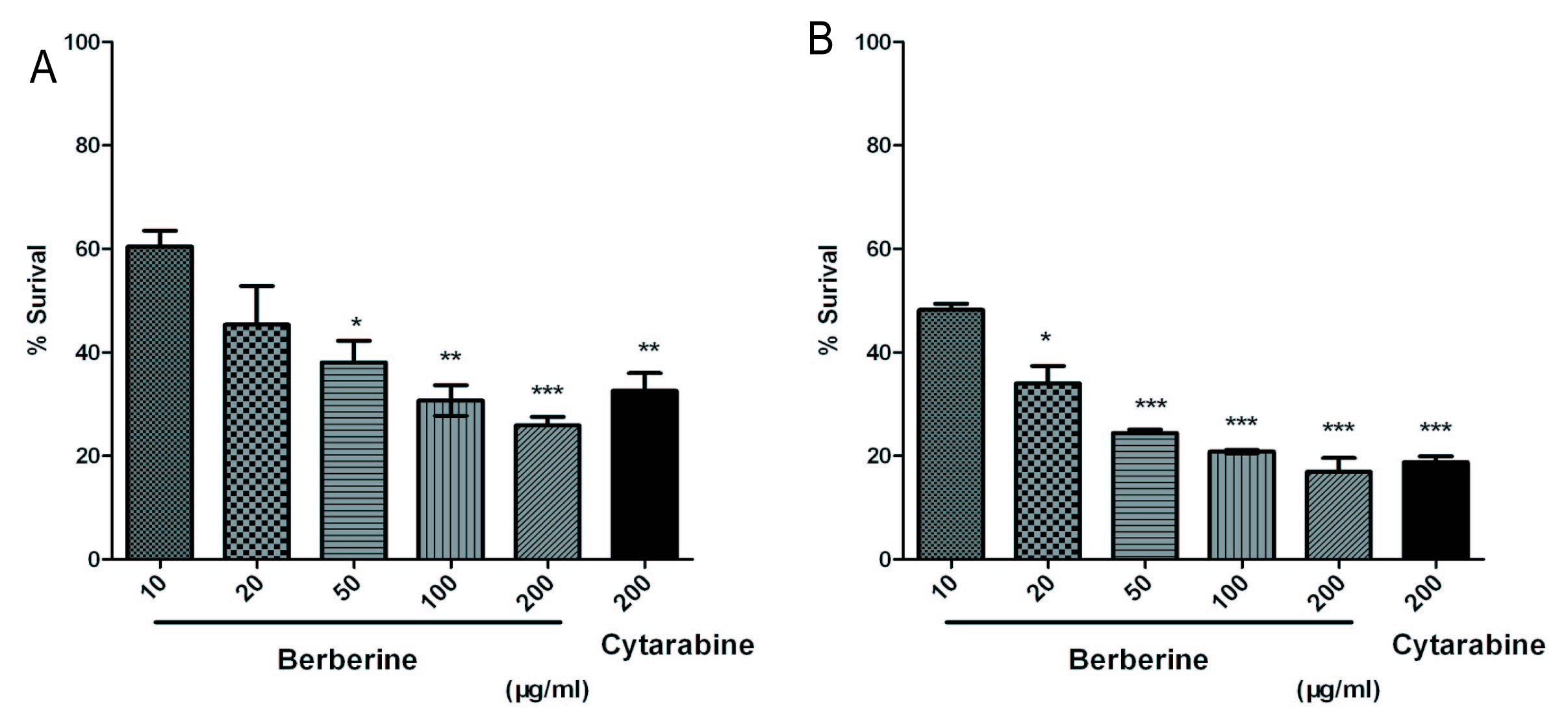


**Figure S2. Effect of a series of berberine and cytarabine solutions on the HL-60 cell line** **at** (A) 24 h and (B) 48 h. The data are expressed as the mean ± S.D. (n = 3); ****P* < 0.001, ***P* < 0.01, and **P* < 0.01. Vs: compare with 10μg/ml berberine.
